# The effect of continuous versus intermittent renal replacement therapy on the outcome of critically ill patients with acute renal failure (CONVINT): a prospective randomized controlled trial

**DOI:** 10.1186/cc13188

**Published:** 2014-01-10

**Authors:** Joerg C Schefold, Stephan von Haehling, Rene Pschowski, Thorsten Onno Bender, Cathrin Berkmann, Sophie Briegel, Dietrich Hasper, Achim Jörres

**Affiliations:** 1 Department of Nephrology and Medical Intensive Care, Charité- Universitätsmedizin Berlin, Campus Virchow-Klinikum, Berlin, Germany; 2 Department of Clinical Cardiology, Charité- Universitätsmedizin Berlin, Campus Virchow-Klinikum, Berlin, Germany; 3 Department of Gastroenterology, Charité- Universitätsmedizin Berlin, Campus Virchow-Klinikum, Berlin, Germany

## Abstract

**Introduction:**

Acute renal failure (ARF) requiring renal replacement therapy (RRT) occurs frequently in ICU patients and significantly affects mortality rates. Previously, few large clinical trials investigated the impact of RRT modalities on patient outcomes. Here we investigated the effect of two major RRT strategies (intermittent hemodialysis (IHD) and continuous veno-venous hemofiltration (CVVH)) on mortality and renal-related outcome measures.

**Methods:**

This single-center prospective randomized controlled trial (“CONVINT”) included 252 critically ill patients (159 male; mean age, 61.5 ± 13.9 years; Acute Physiology and Chronic Health Evaluation (APACHE) II score, 28.6 ± 8.8) with dialysis-dependent ARF treated in the ICUs of a tertiary care academic center. Patients were randomized to receive either daily IHD or CVVH. The primary outcome measure was survival at 14 days after the end of RRT. Secondary outcome measures included 30-day-, intensive care unit-, and intrahospital mortality, as well as course of disease severity/biomarkers and need for organ-support therapy.

**Results:**

At baseline, no differences in disease severity, distributions of age and gender, or suspected reasons for acute renal failure were observed. Survival rates at 14 days after RRT were 39.5% (IHD) versus 43.9% (CVVH) (odds ratio (OR), 0.84; 95% confidence interval (CI), 0.49 to 1.41; *P* = 0.50). 14-day-, 30-day, and all-cause intrahospital mortality rates were not different between the two groups (all *P* > 0.5). No differences were observed in days on RRT, vasopressor days, days on ventilator, or ICU-/intrahospital length of stay.

**Conclusions:**

In a monocentric RCT, we observed no statistically significant differences between the investigated treatment modalities regarding mortality, renal-related outcome measures, or survival at 14 days after RRT. Our findings add to mounting data demonstrating that intermittent and continuous RRTs may be considered equivalent approaches for critically ill patients with dialysis-dependent acute renal failure.

**Trial registration:**

NCT01228123, clinicaltrials.gov

## Introduction

Acute renal failure (ARF) requiring intermittent or continuous renal replacement therapy (RRT) significantly affects morbidity and mortality of critically ill patients and constitutes a substantial health care burden [1]. This remains to be the case despite manifold improvements in RRT techniques and after substantial advances in supportive ICU care. Importantly, development of ARF constitutes an independent risk factor for death in the ICU, and data indicate that early induction of RRT significantly improves the prognosis of affected patients [1-4].

Continuous RRT (CRRT) is widely used in ICUs and is often viewed as the preferable approach in critically ill ARF patients. It remains, however, unclear whether the choice of initial RRT modality may affect patient outcomes, as only few prospective randomized controlled trials (RCTs) have directly compared the different approaches [5-10]. Often these studies were, however, either small and/or had methodologic or randomization problems. In addition, heterogeneous patient cohorts were included (surgical/medical) and different CRRT modalities (convective, diffusive, or both) applied. Only two larger multicenter RCTs directly compared IHD and CVVHD [10] or IHD and CVVH [7]. On the whole, meta-analysis of available data did not demonstrate a general survival benefit for either strategy [3,11-15]. Even fewer data are available regarding end points such as time to hospital/ICU death or discharge, survival at 14 days after RRT, or specific renal-related outcome measures.

To identify an RRT option of choice would potentially have a major impact on clinical procedures. This might be of particular importance in sepsis-induced renal injury [16] and might also affect treatment costs [17]. We therefore embarked on a single-center prospective randomized controlled trial (“CONVINT”) that set out to elucidate further the potential impact of initial choice of RRT modality by studying a cohort of 252 critically ill patients with ARF in medical ICU of a tertiary care academic center.

## Materials and methods

### Design, study patients, and inclusion/exclusion criteria

Patients with ARF requiring RRT treated at the medical intensive care units (ICUs) of the Charité University Hospital, Campus Virchow-Klinikum, Department of Nephrology and Medical Intensive Care, Berlin, Germany, were included in this randomized controlled trial (CONtinuous Vs. INTermittent RRT on the outcome of critically ill patients with ARF trial; CONVINT). CONVINT was performed from January 2002 until October 2007. A single-center prospective randomized controlled open-label trial design applied.

During the recruitment period, adult (>18 years) patients with ARF requiring RRT were eligible for inclusion in CONVINT. Need for RRT was defined as presence of at least one of the following criteria: (a) clinical symptoms of uremia (that is, gastrointestinal symptoms: (for example, nausea, vomiting, diarrhea not explained otherwise; or neurologic symptoms: mental confusion, severe weakness, seizures/coma not explained otherwise; or evidence of pericardial effusion); (b) persisting oliguria (urinary excretion rate <0.5 ml/kg/min for >12 hours), or anuria (anuria for >12 hours or <0.3 ml/kg/h for >24 hours), despite adequate fluid management; (c) fluid overload not responding to diuretic treatment; (d) blood urea nitrogen (BUN) levels >100 mg/dl or creatinine clearance <0.1 ml/ kg of body weight/min; (e) severe metabolic acidosis (pH <7.2) not responding to conventional treatment; and (f) hyperkalemia not responding to conservative treatment.

Patients were excluded when any of the following criteria were met: (a) preexisting chronic renal failure with serum creatinine >3 mg/dl or patients receiving chronic dialysis; (b) kidney-transplant recipients; (c) patients not requiring ICU treatment; (d) denial of written informed consent (patient, legal representative, or legal proxy). The study was approved by the local Ethics Committee (Ethikkommission der Charité-Universitätsmedizin Berlin) and was designed in adherence to the Declaration of Helsinki. Informed consent to participate in the trial was obtained from the patient or legal representative. In case a patient was unable to give informed consent and no legal representative was available, the Ethics Committee approval permitted immediate inclusion of the patient in the trial and to obtain informed consent subsequently, that is, as soon as a legal representative (next of kin without formal rights of representation not sufficient) was installed or the patient regained ability to give informed consent. Data of patients who died before written informed consent was obtained remained in the evaluation. Before randomization, next of kin were always asked if they agreed to including the patient in the study and using the data for future publication. If they declined, or only expressed concerns regarding potential objections the patient himself might have against study participation/publication of data, this patient was not included in the first place.

### Randomization procedure and treatment protocol

After assessment of inclusion and exclusion criteria, patients were allocated to the respective study groups (group 1, IHD; group 2, CVVH) at study day 0 (day of randomization; 1). To assure concealment of allocation, an independent external telephone (computer-based) randomization procedure provided by the Department of Biometry and Medical Documentation, University of Ulm, Ulm, Germany, was used (permutated blocks of four).

IHD (AK100/AK200; Gambro, Lund, Sweden) was performed by using polysulfone synthetic membranes. Standard treatment dose was daily IHD with 4 hours of hemodialysis at a blood flow of 200 to 250 ml/min. A dialysate flow at 500 ml/min, volumetric UF-control, water purified by reverse osmosis, and bicarbonate dialysate was used. Postdilutional CVVH (BM11/BM14; Baxter Medical, Deerfield, IL, USA, or Multifiltrate, Fresenius Medical Care, Bad Homburg, Germany) was applied 24 hours daily by using a polysulfone synthetic membrane, blood flow of 200 ml/min, prescribed filtration rate of 35 ml/ kg of body weight/hour, by using bicarbonate-buffered substitution fluids. General treatment target in both groups was absence of any criteria for acute RRT and a target-time-averaged serum urea of 100 to 150 mg/dl every 72 hours (mean pre + post + pre in IHD; not intended as an outcome measure). CVVH membranes were exchanged every 24 hours in patients with severe sepsis/septic shock. In all other patients, membranes were exchanged every 48 hours. As defined in the study protocol, patients in both study groups were allowed to be switched to the respective other RRT modality in cases of significant medical reasons (that is, either in cases of severe RRT-modality-associated complications or whenever the other modality should not be withheld for significant medical reasons). In such cases, switching of the respective RRT modality was performed by the attending ICU physician. Typical reasons for switching of RRT modality were as follows IHD to CVVH: continuous severe hypotension with requirement of advanced inotropic support or impossibility of maintaining fluid homeostasis. CVVH to IHD: patient mobilization, impossibility of maintaining electrolyte/ acid base homeostasis, impossibility of delivering an adequate dialysis dose, impossibility of performing CVVH without anticoagulation in patients with bleeding complications/repeated filter clotting. Number of changes in RRT modality and respective reasons were recorded.

### Clinical and laboratory follow-up of study patients, outcome measures

Baseline demographics (including hospital/ICU admission day), baseline laboratory data (including baseline blood-gas analyses, urinary output), concomitant diagnoses, and reasons for ARF were recorded. For assessment of clinical disease severity over time, the following clinical scoring systems were used: Sepsis-related Organ Failure Assessment (SOFA) [18], and Therapeutic Intervention Scoring System (TISS)-28 [19]. Acute Physiology And Chronic Health Evaluation (APACHE) II score [20] and Simplified Acute Physiology Score (SAPS)-2 [21] were recorded at baseline. Data on respective RRT treatments, changes in RRT modality, necessity for mechanical ventilation or vasopressor support, routine laboratory data (daily until study day 10 and at days 15, 21), and outcome-related data were recorded daily. Patients were followed up until 14 days after RRT, withdrawal of consent, or death. Follow-up was performed by using electronic hospital data-management systems or telephone follow-up.

The primary outcome measure of CONVINT was survival at 14 days after end of RRT. Secondary outcome measures included 14-day-, 30-day-, all cause intrahospital mortality, days until death (on ICU), days in ICU/hospital, days on RRT/dialysis-free days, total days on vasopressors/mechanical ventilation, and course of disease severity (that is, clinical scores).

### Statistical analyses

The initial power calculation aimed at inclusion of *n* = 200 patients per arm (the OR of failure (that is, primary end point) between the study groups can be given as the probability (true logOR is between estimated logOR ± 0.45) is at least 0.95, under the assumption that failure rates of the therapies may vary within 40% to 80%. No hypothesis of superiority of one of the methods studied, nor equality between the methods, was initially anticipated. Thus, estimation of effects was the focal point. CONVINT was terminated after inclusion of *n* = 252 patients in October 2007 after substantial treatment-protocol changes due to replacement of respective RRT equipment provided by the university hospital (change in contract; machines provided by different supplier; please also refer to Discussion section). Data from two of the 252 included patients were lost to follow-up and could not be included in the final analysis. One patient/proxy per study group withdrew consent (data until withdrawal entered the analysis). With a sample size of 125 patients per group, a precision of log OR ± 0.50 is achieved.

As defined in the study protocol, the sample for the statistical analysis consists of the intention-to treat (ITT) population. Patients were evaluated in the group to which they had been randomly allocated. An explorative PP analysis was performed. Subgroup analyses of patients with need for advanced inotropic support (norepinephrine dose of >0.3 μg/kg/min at any time during the study interval; overall sample group) was performed to identify potential outcome differences between the study groups. Switches from one RRT modality to the other were evaluated as a secondary end point. Statistical methods included Student paired and unpaired *t* test and χ^2^ test, as appropriate. Kaplan-Meier survival estimates were constructed for illustrative purposes. Data were checked for normal distribution by using the Kolmogorov-Smirnov test. Results are reported as means ± standard deviations (SD), if not indicated otherwise. Significance was assigned when *P* < 0.05. Statistical analyses were performed by using MedCalc 12.0.1 software (MedCalc Software, Mariakerke, Belgium).

## Results

### Baseline demographics and study groups

**Figure 1:**
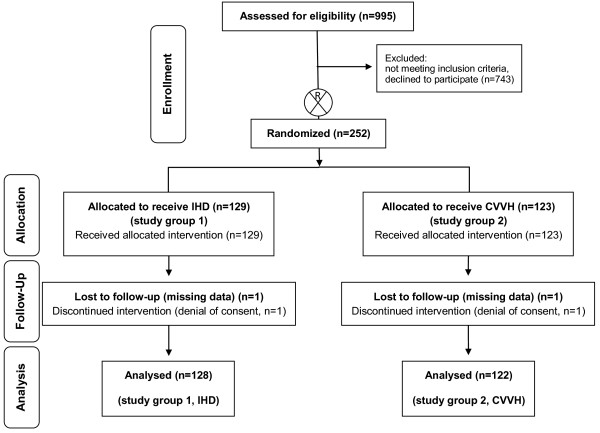
Study flow chart.

Data from *n* = 128 patients (group 1, IHD) and *n* = 122 (group 2, CVVH) patients were analyzed (Figure 1). At baseline, no statistically significant differences between the study groups were noted regarding demographics, days on ICU until study inclusion, disease severity (as assessed by APACHE II, SOFA, SAPS-2, and TISS-28 scores), key physiological and hemodynamic indices (except diastolic blood pressure), renal function, and acid/base balance (Table 1). Concomitant illness was recorded in both study groups. The number of patients with advanced vascular disease, congestive heart failure, chronic obstructive pulmonary disease, and metabolic disease (including diabetes) was not different between the two study groups (Table 1). Major reasons for development of ARF were severe sepsis/septic shock (both groups >65% of study patients at baseline) or cardiogenic shock (please also refer to Table 1). At baseline, >80% of study patients in both groups required both mechanical ventilation and vasopressor support (Table 1) and thus suffered from multiple organ failure. Main underlying cause for developing severe sepsis/septic shock was pneumonia (*n* = 33 patients in group 1 versus *n* = 25 patients in group 2, n.s.). Moreover, 22 patients were initially diagnosed with Acute Respiratory Distress Syndrome (ARDS) (group 1, *n* = 10; group 2, *n* = 12; *P* = n.s.). Peritonitis was present in *n* = 5 (group 1) versus *n* = 5 (group 2) patients (*P* = n.s.). In addition, *n* = 9 (group 1) versus *n* = 8 patients (group 2) were diagnosed with severe pancreatitis. At baseline, no difference in vasopressor need or need for mechanical ventilation was noted (Table 1).

**Table 1:** Baseline demographics/disease severity, and concomitant diseases

		**iHD group**	**CVVH group**	***P *** **value**
		**(*n*** **= 128)**	**(*n*** **= 122)**	
Age (years)		60.8 ± 13.4	62.3 ± 14.5	0.41
Gender (male)		81 (63.3%)	75 (61.5%)	0.97^a^*
Major category (nr. of patients)	Medical	120 (93.8%)	115 (94.3%)	0.95^a^*
	Postsurgery	8 (6.3%)	5 (4.1%)	0.66^a^
	Posttrauma	0	2 (1.6%)	0.46^a^
Body weight (kg)		82.1 ± 22.8	86.1 ± 25.2	0.28
Temperature (°Celsius)		37.5 ± 1.2	37.5 ± 1.3	0.74
Hemoglobin (mg/dl)/(hematocrit) (%)		10.6 ± 1.7	10.4 ± 1.7	0.30
		[31.7 ± 5.8]	[31.4 ± 5.4]	[0.72]
Serum creatinine (mg/dl)		3.64 ± 2.3	3.57 ± 1.9	0.81
Serum urea (mg/dl)		159.7 ± 86.5	156.7 ± 77.1	0.77
Serum potassium (mM)		4.66 ± 0.8	4.65 ± 0.9	0.91
Baseline blood pH		7.32 ± 0.2	7.32 ± 0.1	0.82
Baseline HCO_3_^–^		22.6 ± 6.8	21.8 ± 5.4	0.34
Urine output (within 24 hours before randomization; ml)		927.1 ± 1318.4	708.5 ± 937.8	0.10
Days in ICU until randomization		1.0 [0–3.0]^b^	1.0 [0–2.3]^b^	0.82
Days from ICU admission until start of RRT		1.0 [0–4.0]^b^	1.0 [0–3.0]^b^	0.55
Need for mechanical ventilation (at study day 1)		113 (88.3%)	103 (84.4%)	0.88
paO_2_/FiO_2_ ratio (in all patients at study day 1)		197.3 ± 107.9	208.7 ± 106.8	0.45
Need for (any) vasopressor (at study day 1)		104 (81.2%)	106 (86.9%)	0.79^a^*
Key hemodynamic variables (at study day 1)	Heart rate (/min)	104.0 ± 26.1	104.7 ± 20.9	0.81
	Systolic blood pressure (mm Hg)	111.6 ± 22.5	109.8 ± 19.4	0.51
	Diastolic blood pressure (mm Hg)	56.4 ± 11.1	53.0 ± 13.6	0.04
	Central venous pressure (cmH_2_O)	14.7 ± 5.3	14.2 ± 5.41	0.52
Reason for ARF/need for RRT (number of patients)	Cardiogenic failure/ shock	26 (20.3%)	20 (16.4%)	0.61^a^*
	Sepsis-induced	85 (66.4%)	85 (69.7%)	0.89*
	Hemorrhagic	2 (1.6%)	3 (2.5%)	0.96*
	No shock present	7 (5.5%)	5 (4.1%)	0.85*
	Obstruction-induced	0	3 (2.5%)	0.24*
	Unknown	8 (6.3%)	9 (7.4%)	0.94*
APACHE-II score		28.5 ± 7.9	28.8 ± 9.6	0.79
SAPS-II score		66.1 ± 18.1	63.8 ± 17.6	0.34
SOFA score		13.2 ± 3.9	13.0 ± 4.0	0.66
TISS-28 score		45.0 ± 10.3	47.1 ± 10.2	0.11
Concomitant disease(s) (number of patients; multiple possible)	Atherosclerosis/ischemic HF/AMI	59 (46.1%)	61 (50.0%)	0.80*
	Congestive heart failure	19 (14.8%)	20 (16.4%)	0.91*
	Arterial hypertension	41 (32.0%)	33 (27.1%)	0.61*
	Obstructive pulmonary disease	15 (11.7%)	13 (10.7%)	0.97*
	Diabetes mellitus	22 (17.2%)	18 (14.7%)	0.78*
	Neurologic/psychiatric/post-stroke	27 (21.1%)	29 (23.8%)	0.80*
	Malignancy (solid/hematologic)	30/23	17/22	0.15*
		(23.4%/17.9%)	(13.9%/18.0%)	(0.88)*
	Pancreatitis	9 (7.0%)	8 (6.5%)	0.91*
	Postresuscitation	5 (3.9%)	10 (8.2%)	0.28*
	Posttransplantation	8 (6.3%)	7 (5.7%)	0.92*
	Chronic viral infection	6 (4.7%)	4 (3.3%)	0.82*

Data are given as means ± SD, if not indicated otherwise. Independent samples *t* test, except *a (chi-square), b (median, IQR).

### Follow-up of ARF, need for dialysis, and renal-related outcome measures

The time course of clinical and laboratory data is shown in Table 2. Time until dialysis, days on RRT, dialysis-free days (all n.s.; Tables 1 and 3), as well as course of urinary output, serum creatinine, and serum urea concentrations were not different between the two groups. No differences were noted in serum creatinine at hospital discharge or total fluid balance (Table 3). In the IHD group, the mean blood-flow rate was 222.9 ± 30.4 ml/min, and mean dialysate flow rate was 491.4 ± 6.23 ml/min. Mean duration of IHD was 215.4 ± 82.3 min/session. In the CVVH group, mean applied dose (that is, filtration rate was 30.9 ± 7.0 ml/kg body weight/hour (88% of prescribed dose). Mean blood flow rate in the CVVH group was 188.7 ± 29.4 ml/min. Mean daily ultrafiltration rates were 1.24 ± 0.9 kg (IHD group) and 1.25 ± 1.2 kg (CVVH group) (*P* = 0.87), respectively (means ± SD are given). Anticoagulation was used in 72.8% (IHD) and 74.4% (CVVH) of sessions (unfractionated heparin used in 99.4% versus 97.8% of sessions, respectively). Hirudine derivates or citrate/calcium were used in the remaining cases. Mean dose of unfractionated heparin was 682.8 ± 357 i.U./hour (IHD group) versus 781.6 ± 497 i.U./hour (CVVH group) (*P* = 0.0001).

**Table 2:** Course of disease severity, physiological, and laboratory indices

	**Range, units**	**Group**	**Day 1**	**Day 3**	**Day 5**	**Day 7**	**Day 10**	**Day 15**	**Day 21**	***P *** **value**
										**Day 1 (day 10)**
SOFA score	-	IHD	13.3	13.0	11.5	10.1	7.6	8.3	5.8	n.s. (n.s.)
			±3.7	±5.1	±4.6	±5.2	±5.0	±4.3	±0.8	
		CVVH	13.0	12.2	10.6	9.5	8.2	8.4	4.7	
			±4.0	±3.9	±4.2	±5.2	±5.1	±4.4	±7.2	
TISS-28 score	-	IHD	45.3	42.0	41.2	37.8	29.9	35.8	37.6	n.s. (n.s.)
			±9.5	±9.1	±9.7	±8.0	±9.0	±6.1	±5.5	
		CVVH	46.8	43.3	39.9	37.3	33.8	40.4	25.3	
			±10.2	±8.3	±9.1	±12.3	±10.8	±7.9	±17.2	
Serum creatinine	<1.20 mg/dl	IHD	3.68	3.29	3.17	2.82	2.32	2.54	2.70	n.s. (n.s.)
			±1.86	±2.59	±1.61	±1.51	±1.40	±1.40	±1.54	
		CVVH	3.74	2.18	2.31	2.08	1.93	1.85	1.86	
			±1.88	±1.19	±1.36	±1.40	±1.34	±1.42	±1.52	
Serum urea	14-46 mg/dl	IHD	164.8	121.5	125.4	124.9	105.9	120.4	117.6	n.s. (n.s.)
			±83.4	±55.8	±50.0	±48.9	±53.9	±48.9	±60.4	
		CVVH	155.7	93.4	105.1	107.5	94.4	100.9	94.4	
			±70.0	±44.3	±53.2	±48.9	±48.3	±53.3	±65.7	
Urinary output (24 hours)	>500 ml	IHD	922	944	914	1,331	1,714	1,376	3,570	n.s. (n.s.)
			±1,299	±1,464	±1,415	±1,624	±1,338	±1,508	±3,614	
		CVVH	649	823	1,402	1,645	1,567	1,591	1,964	
			±948	±1,323	±1,785	±1,636	±1,474	±2,093	±757	
pH	7.35-7.45	IHD	7.35	7.37	7.38	7.37	7.39	7.36	7.39	n.s. (n.s.)
			±0.1	±0.1	±0.1	±0.1	±0.1	±0.1	±0.1	
		CVVH	7.36	7.37	7.40	7.38	7.37	7.39	7.39	
			±0.1	±0.1	±0.1	±0.2	±0.1	±0.1	±0.2	
Heart rate	80-100 bpm	IHD	104.0	93.8	90.9	92.9	86.9	85.5	86.5	n.s. (n.s.)
			±26.1	±23.3	±20.3	±17.0	±16.4	±19.9	±12.9	
		CVVH	105.2	93.6	90.0	87.5	89.7	93.1	96.5	
			±21.3	±22.3	±20.4	±19.5	±18.1	±17.2	±15.4	
Mean arterial pressure	50-80 mm Hg	IHD	73.3	76.5	76.9	82.2	78.1	83.3	70.0	n.s. (n.s.)
			±16.5	±17.6	±15.9	±16.7	±17.2	±11.9	±15.1	
		CVVH	72.0	76.2	76.6	77.7	81.2	75.5	75.3	
			±14.1	±13.8	±12.7	±12.4	±14.9	±25.9	±16.5	
Central venous pressure	8-12 mm Hg	IHD	14.7	12.9	12.2	12.2	11.1	10.7	12.9	n.s. (n.s.)
			±5.3	±4.9	±3.9	±4.8	±3.9	±3.4	±2.94	
		CVVH	14.3	13.6	11.8	10.9	12.3	11.5	11.9	
			±5.4	±5.6	±4.2	±3.8	±4.6	±3.6	3.0	
Temperature	36.5°C-37.5°C	IHD	37.5	37.4	37.2	37.4	37.2	37.7	37.7	n.s. (n.s.)
			±1.48	±1.03	±1.3	±0.65	±1.6	±0.67	±0.7	
		CVVH	37.5	37.1	37.1	37.1	37.2	37.1	37.7	
			±1.30	±1.16	±0.94	±0.90	±0.91	±1.05	±1.02	
C-reactive protein	<0.50 mg/dl	IHD	18.0	15.6	10.5	10.5	8.3	7.14	11.7	n.s. (n.s.)
			±10.0	±10.1	±8.3	±7.7	±6.7	±4.95	±8.25	
		CVVH	18.8	15.9	10.1	9.4	10.0	12.6	10.8	
			±10.3	±9.5	±8.55	±7.2	±7.1	±7.5	±7.6	

Last column denotes between-group *P* value (independent-samples *t* test) at study days 1 and 10. Means ± SD are given.

**Table 3:** Clinical outcome of study patients

		**iHD group**	**CVVH group**	***P *** **value**
		**(*n*** **= 128)**	**(*n*** **= 122)**	
Survival at 14 days after RRT		39.5%	43.9%	0.81^a^
14-day mortality rate		43.6%	37.8%	0.63^a^
30-day mortality rate		52.4%	45.4%	0.60^a^
All-cause intrahospital mortality rate (last contact)		60.3%	54.6%	0.72^a^
Days until death		15.6 ± 44.5	18.5 ± 48.9	0.71
Days until death on ICU		15.5 ± 45.9	18.4 ± 50.0	0.73
Days until hospital discharge (in survivors)		51.2 ± 47.1	48.7 ± 49.7	0.78
Days in ICU		25.2 ± 40.1	22.3 ± 26.1	0.50
Days in hospital		33.9 ± 49.3	32.4 ± 37.4	0.79
Suspected reason for death (multiple possible)	Cardiac failure	31	22	0.42^a^
	Pulmonary failure	39	31	0.59^a^
	Sepsis	56	45	0.55^a^
	CNS	7	6	0.92^a^
	Hemorrhagy	5	4	0.93^a^
	Withdrawal of therapy	4	2	0.74^a^
Days on RRT		17.2 ± 37.1	13.7 ± 17.9	0.35
Dialysis-free days		4.2 ± 9.6	3.1 ± 9.0	0.38
RRT switch (number of patients)		25 (19.5%)	56 (45.9%)	0.002^a^
Number of patients on RRT (% of survivors, days after ICU admission)	At 21 days	20 (32.3%)	20 (29.9%)	0.97^a^
	At 60 days	14 (26.4%)	13 (22.8%)	0.90^a^
Serum creatinine at hospital discharge/last contact (in survivors; mg/dl)		2.18 ± 1.8	2.12 ± 1.7	0.85
Total days on vasopressors		4.3 ± 3.7	4.5 ± 3.7	0.75
Cumulative vasopressor dose (g)	Epinephrine	0.70	0.64	0.96
	Norepinephrine	19.1	18.5	0.30
	Dobutamine	150.2	137.9	0.56
Total days on mechanical ventilation		8.1 ± 8.8	7.2 ± 6.5	0.34
Total fluid balance (L)		20.5 ± 23.2	24.9 ± 28.4	0.19

Between-group *P* values are given. Independent samples *t* test except ^a^(χ^2^). Means ± SD.

As defined in the study protocol, patients in both groups were allowed to cross over to the respective other RRT modality in case of significant medical reasons. Switch of RRT modality was decided by the attending ICU physician in charge. In total, 19.5% patients randomized to receive IHD (group 1) versus 45.9% patients randomized to receive CVVH (group 2) were switched to the respective other modality during the study period. Time from both randomization and ICU admission until switch was not different between the two study groups: days from randomization until switch 4.4 ± 12.0 (group 1) versus 6.2 ± 5.6 (group 2), *P* = 0.37. In the IHD group (group 1), switching of modality was necessary mainly due to reasons related to progressive hemodynamic instability (that is, advanced inotropic support) and/or due to significant fluid overload (16%).

In group 2, switching of modality was mostly indicated because of repeated filter clotting (27%), metabolic reasons (for example, progressive lactic acidosis; 18%), bleeding/discontinuation of anticoagulation required (11%), severe thrombocytopenia (5%), or because of desired mobilization/clinical improvement of study patients.

### Course of disease severity/organ dysfunction, key physiological and laboratory indices

Disease severity was assessed over time by using SOFA and TISS-28 scoring systems, revealing no differences between the study groups (Table 2). Cumulative catecholamine need and total days on vasopressors were not different between the two study groups (data not shown). Key hemodynamic variables (that is, heart rate, mean arterial pressure, and central venous pressure) did not differ between the study groups at randomization, baseline, and during follow-up (Tables 1 and 2). Need for mechanical ventilation was assessed in both study groups. Initial need for mechanical ventilation (Table 1), total days on mechanical ventilation (Table 3), and paO_2_/FiO_2_ (data not shown) did not differ between the study groups. During follow-up, other physiological variables including blood pH, urinary output, temperature, and C-reactive protein were also not different (Table 2).

### Survival at 14 days after RRT, 14-day-, 30-day- and all-cause mortality data

Survival at 14 days after RRT, 14-day-, 30-day- and all-cause intrahospital mortality rates were not different between the study groups in both the ITT and PP populations (all n.s.; survival rates of the ITT population given in Table 3). The univariate OR (for patients in IHD group) for survival at 14 days after end of RRT was 0.84 (95% CI, 0.49 to 1.41; *P* = 0.5). OR (for patients in IHD group) for 14-day mortality was 1.27 (95% CI, 0.76 to −2.12; *P* = 0.36). Missing data (survival at 14 days after RRT) in nine (IHD group) versus eight (CVVH group) patients (that is, 7.0%, n.s.). OR (IHD group) for 28-day mortality was 1.37 (95% CI, 0.82 to 2.27; *P* = 0.22). For patients randomized to receive IHD, the OR for 30-day mortality was 1.32 (95% CI, 0.80 to 2.19; *P* = 0.27), and for all-cause intrahospital mortality, 1.26 (95% CI, 0.76 to 2.10; *P* = 0.37). In the CVVH study group, the survival rate at 14 days after RRT was 4.4% higher, and mortality rates were not significantly reduced by 5.8% (14-day mortality), 7.9% (28-day mortality), 7.0% (30-day mortality), and 5.7% (all-cause intrahospital mortality).

To investigate the potential impact of modality switch on outcome indices, subanalyses were performed comparing patients who switched with those who stayed on the initial therapy, revealing no influence on the primary outcome measure, 14-day mortality, 30-day mortality, or all-cause intrahospital mortality (all *P* = n.s., data not shown).

**Figure 2:**
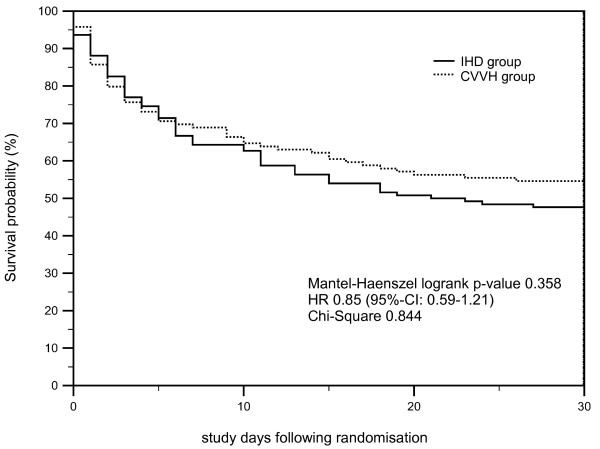
**Kaplan-Meier survival estimates for patients randomized to IHD (full line, *n*** **= 128) and CVVH (dotted line, *n*** **= 122) are illustrated (total study population).** Mantel-Haenszel log-rank *P* value, hazard ratio (HR) including 95% CI and χ^2^ is given.

**Figure 3:**
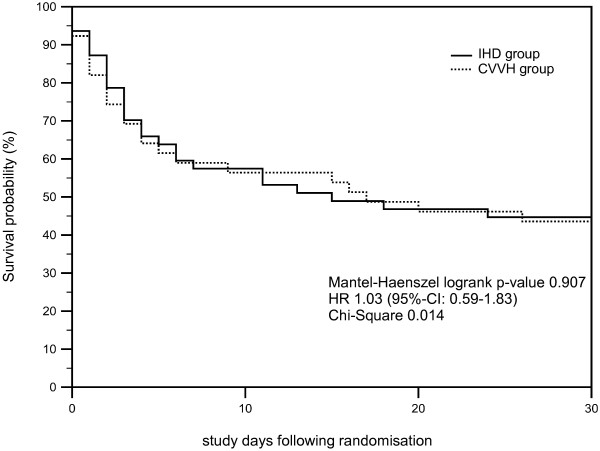
**Kaplan-Meier survival estimates for the subpopulation of patients with high vasopressor need (overall sample; high vasopressor use defined as >0.3 μg/kg/min at any point in time during the study interval) in the IHD (full line, *n*** **= 47) versus CVVH (dotted line, *n*** **= 40) groups are illustrated.** Mantel-Haenszel log-rank *P* value, hazard ratio (HR), including 95% CI and χ^2^ is given.

Moreover, we investigated whether higher norepinephrine need (defined as >0.3 μg/kg/min at any time during the study interval) was associated with different outcomes in the respective study groups. It was noted that, in this subgroup with higher catecholamine need (*n* = 47 in group 1 versus *n* = 40 in group 2), survival at 14 days after RRT (36.2% versus 37.5%; *P* = 0.9), 14-day-, 30-day-, and all-cause mortality rates (65.9% versus 60.0%; *P* = 0.92) were also not different between the study groups. To illustrate survival characteristics, Kaplan-Meier survival estimates were constructed for the total study population (Figure 2) and the subpopulation receiving a higher vasopressor dose (Figure 3).

## Discussion

Acute renal failure (ARF) remains an important determinant for patient outcomes and constitutes a particular burden for healthcare systems worldwide [1]. Here we investigate the potential impact of two of the major RRT modalities (that is, IHD and CVVH) on mortality and renal-related outcome measures in a single-center prospective randomized controlled trial (CONVINT). After inclusion of 252 patients with ARF requiring RRT, we observed no statistically significant differences in 14-day, 30-day, and all-cause mortality, renal-related outcome measures, or survival at 14 days after RRT. Recent meta-analyses call for larger RCTs because of limited sample sizes of previous clinical trials and considerable heterogeneity of respective study populations [3,11-15]. Our data support findings from previous studies that intermittent and continuous RRT (CRRT) modalities may be considered equivalent approaches in a general population of critically ill patients with dialysis-dependent ARF.

Our study population consisted of a cohort of critically ill medical ICU patients with persisting ARF requiring RRT, despite adequate hemodynamic status. At study day 1, the mean APACHE II score in the overall sample was 30.4 ± 7.1. The majority of patients initially presented with established multiple organ failure. The major underlying condition for development of multiple organ failure at baseline was severe sepsis/septic shock. Besides requiring renal support, most patients required mechanical ventilation and vasopressor support also (please refer to Table 1). This may explain the rather high mortality rates observed in our trial (Table 3, Figure 2). In line with previously published larger clinical trials in patients with ARF, severe sepsis/septic shock due to pneumogenic or abdominal sepsis was the major underlying pathology in our study population.

In CONVINT, we deliberately chose to exclude patients with preexisting advanced chronic renal failure (that is, patients with previous serum creatinine values of >3 mg/dl). Patients in CONVINT received daily IHD for at least 4 hours. In CVVH-treated patients prescribed “dose” (that is, filtration rate was 35 ml/kg/h based on the landmark trial by Ronco *et al*. [22] with a delivered dose of 30.9 ± 7.0 ml/kg/hour). This discrepancy is in line with previous publications [23] demonstrating that the applied dose may differ from the prescribed dose in critically ill patients. This must be kept in mind when prescribing CRRT.

Until recently, however, the optimal dose of CRRT in critically ill patients with ARF was controversial. Data from two recent large-scale multicenter RCTs now demonstrate that filtration rates above 25 ml/kg/hour may not further improve the outcome of critically ill patients with ARF [24,25]. Thus, the filtration rate actually applied in CONVINT could be considered adequate on the basis of recent recommendations [26].

As recent guidelines [26] also specifically suggest preferential use of CRRT in hemodynamically unstable patients, we also investigated the effect of IHD versus CVVH in the subgroup of patients with relevant vasopressor requirements. In the overall study population, mortality rates or survival at 14 days after RRT in patients with norepinephrine need of >0.3 μg/kg/min were not different between the two study groups (Figure 3), however, the number of study patients in this subgroup was rather small. This finding should therefore not be used as an argument against the preferential use of CRRT in hemodynamically compromised individuals. As recommended by the KDIGO guideline [26], priority should be given to individualized therapeutic decisions that are made by ICU specialists or consulting nephrologists on the basis of the specific clinical and hemodynamic status of the patient.

A number of limitations of our analysis require discussion. First, the initial power calculation aimed at inclusion of 200 patients per arm. CONVINT was terminated early after inclusion of 252 patients (63% of the targeted population). The reason for early trial termination was a major change in RRT equipment and procedures that was beyond the investigators’ control. With the ICU team confronted with new machinery and new treatment protocols (for instance, automated citrate anticoagulation, preference for HDF instead of HD), it became obvious that this would introduce an unacceptable bias to the trial. The investigators therefore decided to terminate the study prematurely.

After termination of the trial, a retrospective explorative analysis was performed to determine whether the trial might have been able to reach statistical significance regarding the primary end point if continued as planned. This was, however, not the case, as the between-group *P*value for the primary outcome measure would not have reached statistical significance in the unlikely event that survival rates at 14 days after RRT in the 148 remaining cases had differed between groups by an additional 30%. However, by definition, we cannot rule out an effect of underpowering and related beta error on the outcome measures (including renal outcome measures) after early trial termination.

Second, switching of RRT modality in cases of significant RRT modality-related complications or medical reasons was deliberately allowed in the study protocol. This was done for safety reasons and was considered inevitable in this cohort of critically ill patients. Although the substantial number of cross-overs could have influenced our overall findings, differences in both the primary end point and respective mortality rates in the subgroups of patients switched/not switched were not observed.

Third, we present data from a single-center RCT, and the overall trial period was rather long. Thus, the inherent limitations of monocentric trials should be kept in mind when analyzing the data provided. Nevertheless, as therapeutic standards of care were unchanged during the trial period, we believe that the total study interval should be a minor limitation of our analysis.

Fourth, we defined a previous serum creatinine value of >3 mg/dl as an exclusion criterion to exclude patients with advanced chronic renal failure. Conversely, patients with moderate to advanced chronic renal insufficiency could be included in the study and might have influenced our data.

## Conclusions

In conclusion, after recruitment of 252 patients with dialysis-dependent ARF to a prospective single-center randomized controlled trial, we observed no statistically significant differences regarding 14-day-, 30-day-, all-cause intrahospital mortality, renal-related outcome measures, or survival at 14 days after RRT. Moreover, no significant effect of the RRT modality on the percentage of surviving patients still requiring RRT at days 21 or 60 was observed. Our data lend further support to the view that intermittent and continuous RRT may be considered equivalent approaches for a general population of critically ill patients with dialysis-dependent ARF treated in a medical ICU.

## Key messages

• In critically ill patients with dialysis-dependent ARF, statistically significant differences regarding mortality rates, renal-related outcome measures, or the rate of surviving patients at 14 days after end of RRT were not observed between patients randomized to receive either daily iHD or CVVH.

• In the subgroup of patients with higher catecholamine need, survival at 14 days after RRT, 14-day, 30-day, and all-cause mortality rates were also not different between the two RRT modalities (that is, daily iHD versus CVVH).

• Our data support the view that intermittent and continuous RRT may be considered equivalent approaches for a general population of critically ill patients with dialysis-dependent ARF treated in a medical ICU.

## Abbreviations

ARF: Acute renal failure; CVVH: continuous venovenous hemofiltration; iHD: intermittent hemodialysis; KDIGO: Kidney Disease: Improving Global Outcomes; RRT: renal replacement therapy.

## Competing interests

The authors declare that they have no conflict of interest, financially or otherwise, to disclose.

## Authors’ contributions

JCS, SvH, RP, TOB, DH, and AJ designed, supervised, and analyzed all data. JCS, RP, and AJ wrote the manuscript. CB and SB acquired data and analyzed all data. All authors read and approved the final version of the manuscript.
